# Changes in population characteristics and their implication on public health research

**DOI:** 10.1186/1742-5573-4-6

**Published:** 2007-07-10

**Authors:** Ping Du, F Bruce Coles, Patricia O'Campo, Louise-Anne McNutt

**Affiliations:** 1 Division of Epidemiology, New York State Department of Health, Albany, NY, 12237, USA; 2 Department of Epidemiology and Biostatistics, University at Albany, State University of New York, Rensselaer, NY, 12144, USA; 3 Centre for Research on Inner City Health, University of Toronto, St. Michael's Hospital, 30 Bond Street, Toronto, Ontario, M5B 1W8, Canada

## Abstract

Population estimates are generally drawn from one point in time to study disease trends over time; changes in population characteristics over time are usually not assessed and included in the study design. We evaluated whether population characteristics remained static and assessed the degree of population shifts over time. The analysis was based on the New York State 1990 and 2000 census data with adjustments for changes in geographic boundaries. Differences in census tract information were quantified by calculating the mean, median, standard deviation, and the percent of change for each population characteristic. Between 1990 and 2000, positive and negative fluctuations in population size created a U-shaped bimodal pattern of population change which increased the disparities in demographics and socioeconomic status for many census tracts. While 268 (10%) census tracts contracted by 10%, twice as many census tracts (21%, N = 557) grew at least 10%. Notably, the non-Hispanic African-American population grew 10% or more in 152 tracts. Although there were overall reductions in working class and undereducated populations and gains in incomes, most census tracts experienced growing income inequalities and an increased poverty rate. These changes were most pronounced in urban census tracts. Differences in population characteristics in a decade showed growing disparities in demographics and socioeconomic status. This study elucidates that important population shifts should be taken into account when conducting longitudinal research.

## Background

The social environment is increasingly appreciated as an important risk factor for disease, independent of an individual's characteristics [1-14]. To investigate the association between the social environment and health outcomes, area-based population characteristics obtained from the decennial census data are widely utilized in public health research. Typically, the relevant census data is linked to the incidence rate of disease in ecologic studies or modeled in conjunction with individual-level characteristics to estimate the risk of disease. These studies identify certain population characteristics, including socioeconomic status, as important predictors for disease development [3,5,8,9,11-19]. However, in some longitudinal research, population estimates are generally drawn from one point in time to study disease trends over time and study designs do not usually include changes in population composition, despite the possibility that disease trends may be influenced by changes occurring in either the population or the geographic area where people live [20,21].

Assessing population dynamics is critically important when evaluating the etiology of disease trends that are not fully explained by changes in individual risk factors and medical advances. Ecologic studies have demonstrated that changes in area-based socioeconomic characteristics were associated with changes in mortality [22] and tuberculosis incidence [23]. In other research, multilevel analyses which controlled for individual characteristics uncovered significant associations between changes in neighborhood's demographic and socioeconomic characteristics and reproductive outcomes [11,12]. These findings imply a possible causal link between the social environment and the risk of disease. This association may persist because the community context affects knowledge, attitudes, and behaviors for individuals living in the community [24]. Thus, identifying changes in population characteristics of communities may help to understand risk factors that contribute to the development of disease at both individual level and population level.

Shifting population census characteristics have been observed at different geographic levels ranging from the nation to the census block group [22,23,25-29]. Because the census tracts, considered to be "relatively homogeneous units with respect to population characteristics", are commonly used in public health research to approximate "community" or "neighborhood", this study evaluated whether population characteristics remained static, and if not, assessed the degree of changes for some commonly used census variables across census tracts in New York State (NYS) exclusive of New York City (NYC) between 1990 and 2000. Changes across the urban-rural gradient were also studied.

## Analysis

### Data sources

The 1990 and 2000 census data at the census-tract level for NYS exclusive of NYC were obtained from the Neighborhood Change Database (NCDB, ^© ^1996–2003 GeoLytics, Inc., East Brunswick, NJ, U.S.A.) [30]. The 1990 census tracts were standardized and modified to the 2000 boundaries with an advanced geographic information system in order to directly compare population data across censuses. Briefly, the boundaries of the 2000 tracts were overlaid with the 1990 boundaries. The 1990 tract-level variables were then converted to the 2000 tract boundaries based on the proportion of the population in the 1990 block-level data that became part of the 2000 tract [31]. About half (1,242 (46.2%)) of the 2,690 tracts in the 2000 census were redrawn by splitting, merging or regrouping the 1990 census tracts. To avoid unstable population estimates, 68 (2.5%) census tracts were excluded from this study because they had less than 100 persons or less than 30 households in either census year, leaving a total of 2,622 census tracts for analysis. In this study, the census data represented NYS exclusive of NYC and will hereafter be referred to as the "dataset."

The selection of census variables for this study was based on commonly utilized demographic, socioeconomic and housing indicators in previous research [10,17,18,20,32,33], and the definitions for those indicators are presented in Table 1. The Gini coefficient measuring income inequality was calculated based on the distribution of household income [34]. Its value, ranging from 0 to 1, reflects the degree of income inequality: 0 means equal distribution of household income (i.e., every household in the census tract has the same income); and 1 means complete inequality (i.e., one household has all the income, other households earn nothing).

**Table 1:** Definitions for census-based population characteristics

**Characteristics**	**Definitions**
**Demographics**	
Population size	All persons living in a given census tract
Female-headed families	Percent of families maintained by a female householder with no husband present
Male	Percent of male population
Non-Hispanic white	Percent of persons who identify them as White and non-Hispanic origin
Non-Hispanic African-American	Percent of persons who identify them as African-American and non- Hispanic origin
Hispanic	Percent of persons who identify them as "Hispanic" or "Latino"
15–44 age group	Percent of persons who are 15-to-44 years old
Never married	Percent of persons >= 15 years old who are never married
Foreign born population	Percent of persons who are not U.S. citizens at birth
**Socioeconomic status**	
Unemployment rate	Percent of persons >= 16 years old who are in the labor force and unemployed
Working class	Percent of employed persons>= 16 years old in the following occupations: sales; administrative support; service except protective service; precision production, craft, and repair; operators, fabricators, and laborers.
High school dropout rate	Percent of persons 16–19 years old neither enrolled in nor graduated from high school
Below a high school degree	Percent of adults >= 25 years old whose highest level of schooling is less than a 12^th ^graduate
Income (1999 $)	The sum of reported incomes
Per capita income	The average individual income
Median family income	The middle value of income in the family income distribution
Median household income	The middle value of income in the household income distribution
Income inequality (gini coefficient)	Calculated based on the household income distribution using method provided by Shryock [34] with the range of 0–1.
Poverty	The total income for an individual, a family or a household below the federal poverty threshold.
	1989 threshold for an individual: $6,310
	1999 threshold for an individual: $8,499
	1989 threshold for a 4-person family (household): $12,674
	1999 threshold for a 4-person family (household): $17,030
Individuals below poverty	Percent of population below the poverty level
Families below poverty	Percent of families below the poverty level
Households below poverty	Percent of households below the poverty level
Households with interest, dividend, and rental income	Percent of households with interest, dividend, and rental income
**Housing**	
Home ownership	Percent of housing units occupied by owners
Median house value (1999 $)	The middle value of owner-occupied houses
Vacant housing units	Percent of housing units with no one living in
House crowding	Percent of occupied housing unit with > 1 person per room

In the 2000 census questionnaire, individuals could report multiple races whereas, in previous years, only one race category could be chosen. In our dataset, only a small portion of the 2000 census population (1.9%) actually took advantage of this option. In the NCDB, a hierarchy was utilized to convert multiracial categories initiated in the 2000 census to a single race category [31]. By combining information on Hispanic or Latino ethnicity, race was further categorized as non-Hispanic whites, non-Hispanic African-Americans, Hispanics, and others.

To adjust the 1990 incomes for inflation, incomes from the 1990 census (recorded in 1989 dollars) were adjusted to 1999 dollars according to the Consumer Price Index ratio (CPI = 1.344) [35]. The 1990 occupation categories were converted to the 2000 census categories based on conversion factors listed in the 1990–2000 Census Tabulation Crosswalk Template for Occupation [36].

### Statistical analysis

In some studies, changes were quantified by the difference between means of a given characteristic, and in others, the degree of change is quantified by the standard deviation [23,26,28,29]. To examine the overall population shifts between 1990 and 2000, the mean, median and standard deviation (SD) of specific characteristics were calculated for 2,622 census tracts; as in previous research, the difference in means was compared for each characteristic.

The extent of change across individual census tracts was measured for each population characteristic. The percent differences for each census tract were then grouped into 11 categories to graphically depict the relative magnitude of change by characteristic: decrease: >= 10%, 5–<10%, 3–<5%, 1–<3%, and <1%; no change (the difference = 0); increase: <1%, 1–<3%, 3–<5%, 5–<10%, and >= 10%. For incomes and house value the categories of change utilized for descriptive data presentation were: decrease: >= $10,000, $5,000–<$10,000, $3,000–<$5,000, $1,000–<$3,000, <$1,000; no change (the difference = 0); increase: <$1,000, $1,000–<$3,000, $3,000–<$5,000, $5,000–<$10,000, and >= $10,000.

Population changes were further assessed according to rural-urban status. The classification of rural-urban status was based on the 1993 rural-urban continuum codes provided by the Economic Research Service and population density [16,37]. Counties were classified as urban counties if they were defined as large metropolitan counties (continuum code 0 or 2) and had more than 60% of the population living in census-defined urbanized areas in 1990. Within these urban counties, census tracts with 5,000 or more people per square mile (PPSM) were defined as "Metro: urban-high population density," tracts with 1,000 to 4,999 PPSM were classified as "Metro: urban-medium population density," and tracts with less than 1,000 PPSM were categorized as "Metro: urban-low population density." Census tracts located within the rest of the metropolitan counties (continuum code 1 or 3) were classified as "Metro: non-urban," and census tracts located outside metropolitan counties (continuum codes 4–9) were defined as "non-Metro: rural."

All data were analyzed using SAS ^®^, Version 8.2, software (SAS Institute Inc., Cary, NC, U.S.A.)

## Results

In 2000, the Census Bureau reported that there were 10,968,179 people residing in NYS exclusive of NYC, a 2.8% increase from 10,667,639 in 1990. The average population size in census tracts was 4,165 people in 1,539 households. Based on the criteria for classifying the rural-urban status, 740 census tracts were defined as "Metro: urban-high population density," 739 were "Metro: urban-medium population density," 408 were "Metro: urban-low population density," 347 were "Metro: non-urban," and 388 census tracts were defined as "non-Metro: rural."

Table 2 presents population characteristics in the 1990 and the 2000 censuses. The differences in means reflect the average change in population characteristics across census tracts. Regarding the racial-ethnic distribution, non-Hispanic African-Americans, Hispanics, and foreign-born populations gradually grew during the decade. An increase in the mean percent of female-headed families was also observed, especially those with children. Individuals 15–44 years of age continued to account for the largest portion of the total population, however this group decreased in relative size due to a large increase in the number of individuals aged 45 years and older. The male population and the never married population remained relatively stable over time.

**Table 2:** Census-tract level population characteristics in NYS exclusive of NYC, 1990 and 2000

	**1990 census**		**2000 census**		
		
**Characteristics**	**Mean** **(Median)**	**SD**^a^	**Mean** **(Median)**	**SD**^a^	**Difference** **In Mean**
Population size	4,044	1,541	4,165	1,697	121
	(3,967)		(4,014)		
Female-headed families, %	15.5 (12.0)	11.1	17.8 (13.6)	12.3	2.3
Male, %	48.4 (48.5)	3.21	48.6 (48.6)	3.40	0.2
Non-Hispanic white, %	87.0 (94.4)	20.0	81.7 (91.2)	23.1	-5.3
Non-Hispanic African-American, %	7.20 (1.25)	16.5	9.01 (1.98)	17.4	1.8
Hispanic, %	3.73 (1.75)	6.36	6.07 (2.88)	9.32	2.3
15–44 age group, %	46.0 (45.1)	6.76	41.8 (40.9)	7.26	-4.2
Never married, %	27.8 (25.3)	9.30	27.8 (24.8)	9.50	0
Foreign born population, %	6.99 (5.17)	6.45	8.54 (5.64)	8.65	1.5
Unemployment rate, %	5.98 (4.99)	3.80	5.79 (4.48)	4.90	-0.1
Working class, %	61.1 (62.3)	9.57	56.2 (57.3)	10.8	-4.9
High school dropout rate, %	8.95 (6.78)	9.10	7.96 (5.47)	9.38	-1.0
Below a high school degree, %	21.6 (20.4)	10.7	16.9 (15.2)	10.0	-4.7
Per capita income (1999 $)	21,203	10,027	23,448	11,008	2,245
	(18,997)		(20,911)		
Median family income (1999 $)	56,871	24,093	58,741	26,571	1,870
	(52,871)		(53,391)		
Median household income (1999 $)	48,571	24,731	50,846	24,799	2,275
	(42,907)		(45,703)		
Gini coefficient	0.3790	0.0525	0.4050	0.0584	0.026
	(0.3746)		(0.3984)		
Individuals below poverty, %	9.67 (6.21)	10.0	11.0 (7.52)	10.5	1.3
Families below poverty, %	7.19 (4.21)	8.74	8.30 (5.17)	9.30	1.1
Household below poverty, %	9.59 (6.66)	9.49	10.5 (7.42)	9.80	0.9
Household with interest, dividend, and rental income, %	47.6 (48.1)	14.0	41.3 (41.4)	14.3	-6.3
Home ownership, %	69.5 (76.0)	21.1	69.3 (75.9)	21.7	-0.2
Median house value, 1999 $	159,255	116,794	145,779	117,774	-13,476
	(117,061)		(102,450)		
Vacant housing units, %	8.89 (5.05)	11.2	9.11 (5.27)	10.7	0.2
House crowding, %	2.19 (1.29)	3.39	2.77 (1.55)	4.08	0.6

^a^SD: standard deviation.

Socioeconomic disparities appeared to grow during the decade. Both average incomes and poverty rates increased, resulting in an increasing Gini coefficient, a measure of income inequality. While professional jobs increased, minimal decreases were seen in unemployment or poor housing conditions. The mean house value dropped by more than $13,000 (8.5%) from 1990 to 2000.

### Changes in population characteristics in 1990–2000 at the census tract level

**Figure 1:**
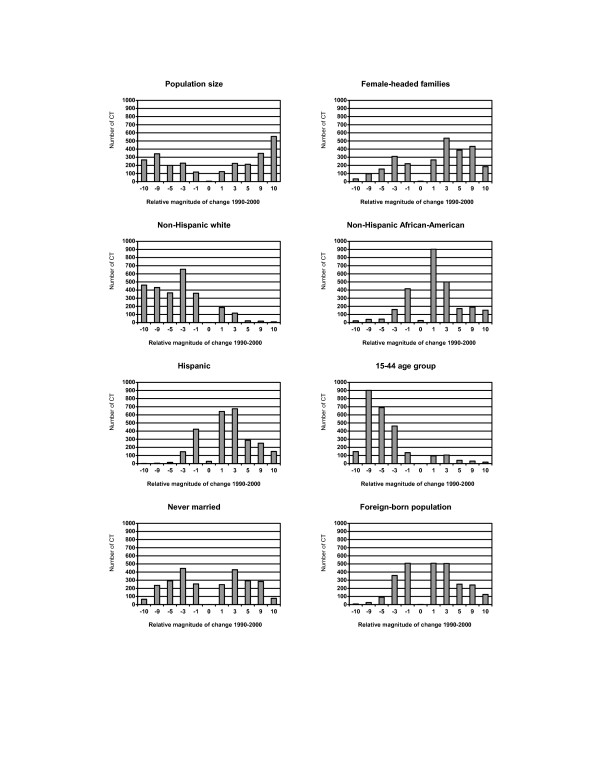
Distribution of changes in population demographic characteristics across 2,622 census tracts (CT) in NYS exclusive of NYC, 1990–2000. **Relative magnitude of change 1990–2000: **-10: decrease >= 10% -9: decrease 5–<10% -5: decrease 3–<5% -3: decrease 1–<3% -1: decrease <1% 0: no change 1: increase <1% 3: increase 1–<3% 5: increase 3–<5% 9: increase 5–<10% 10: increase >= 10%

Some population demographic characteristics within individual census tracts changed considerably between 1990 and 2000 (Figure 1). While overall population growth in the state was modest, the census tract analysis revealed a U-shaped pattern of population change distribution. Population size increased by more than 10% in 557 (21%) census tracts, while 268 (10%) census tracts experienced a decline of at least 10% of their population. The distribution of changes in the female-headed families skewed to the left because more census tracts had increased numbers of female-headed families. An increase in the percent of non-Hispanic African-American population was evident across most census tracts, and 152 (6%) tracts experienced at least a 10% increase. Although a decrease in the non-Hispanic African-American population occurred among 679 tracts, the reduction was generally less than 3%. Changes in the Hispanic and foreign-born population followed a similar pattern. In contrast, the percent of non-Hispanic whites decreased in many census tracts. Population aging was apparent in the upward migration from the 15–44 year age group. A bimodal pattern indicated both declining and growing of the never married population across census tracts.

**Figure 2:**
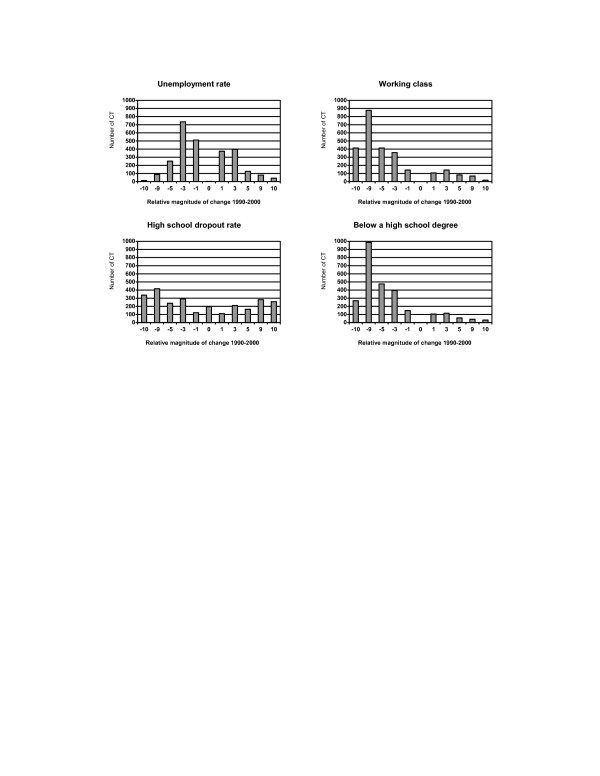
Distribution of changes in population socioeconomic status (occupation and education) across 2,622 census tracts (CT) in NYS exclusive of NYC, 1990–2000. **Relative magnitude of change 1990–2000**:  -10: decrease >= 10% -9: decrease  5–<10% -5: decrease 3–<5% -3: decrease 1–<3% -1: decrease <1% 0: no change  1: increase <1% 3: increase 1–<3% 5: increase 3–<5% 9: increase 5–<10% 10:  increase >= 10%

**Figure 3:**
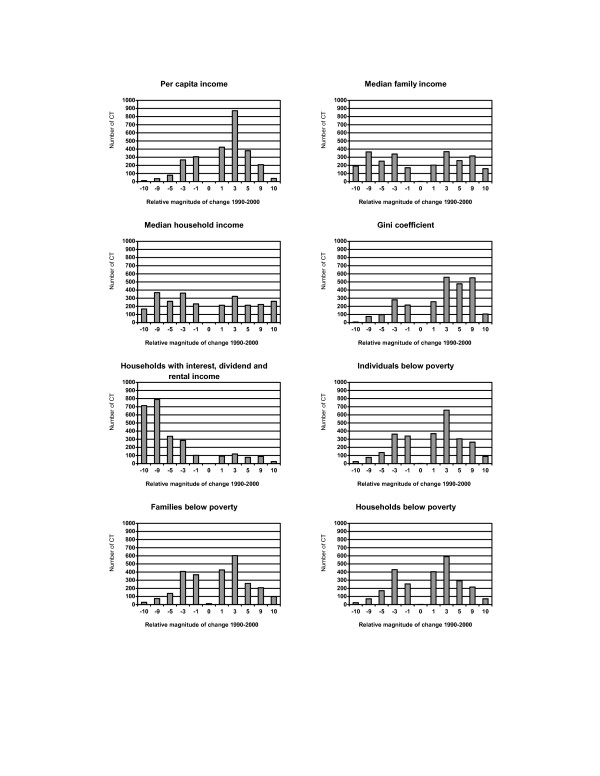
Distribution of changes in population socioeconomic status (income and poverty) across 2,622 census tracts (CT) in NYS exclusive of NYC, 1990–2000. **Relative magnitude of change 1990–2000: **-10: decrease >= 10% -9: decrease 5–<10% -5: decrease 3–<5% -3: decrease 1–<3% -1: decrease <1% 0: no change 1: increase <1% 3: increase 1–<3% 5: increase 3–<5% 9: increase 5–<10% 10: increase >= 10% **Relative magnitude of change 1990–2000 for incomes and median house value: **-10: decrease >= $10,000 -9: decrease $5,000–$10,000 -5: decrease $3,000–<$5,000 -3: decrease $1,000–<$3,000 -1: decrease <$1,000 0: no change 1: increase <$1,000 3: increase $1,000–<$3,000 5: increase $3,000–<$5,000 9: increase $5,000–<$10,000 10: increase >= $10,000.

Socioeconomic status demonstrated greater disparities across census tracts (Figure 2 and Figure 3). A reduction in the unemployment rate was seen among 60% (1,600) of 2,622 census tracts, but the degree of change was usually less than 3% (Figure 2). The percent of the working-class population declined among most census tracts, and 16% (414) of 2,622 tracts had at least a 10% drop due to an increase in professional employment. In general, an improvement in education was observed among most census tracts with reductions in the high school dropout rate. However, a total of 257 (10%) census tracts exhibited a greater than 10% increase in the high school dropout rate. While an increase in the per capita income was observed for 73% (1,923) of census tracts, changes in the median family income and the median household income were quite variable (Figure 3). Worthy of note, poverty rates and income inequality worsened among most census tracts. A great reduction was observed in the percent of households having interest, dividend, and rental income across census tracts.

**Figure 4:**
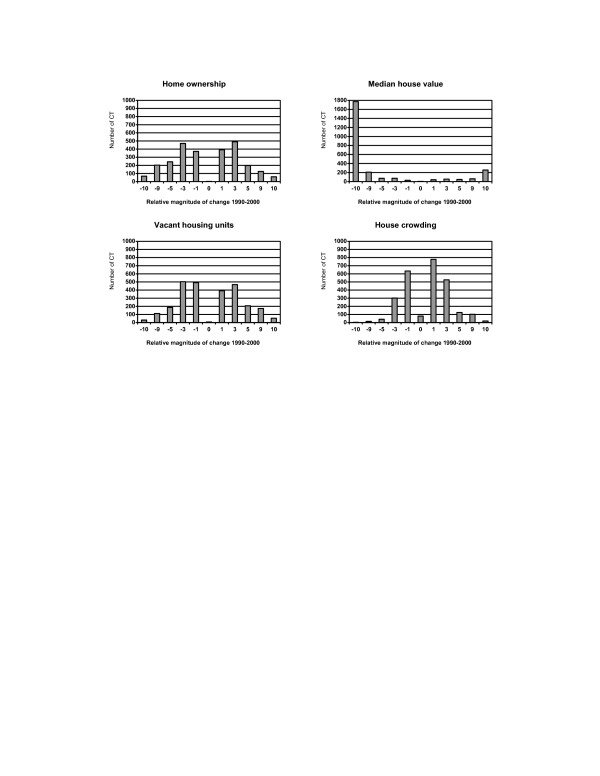
Distribution of changes in population housing conditions across 2,622 census tracts (CT) in NYS exclusive of NYC, 1990–2000. **Relative magnitude of change 1990–2000: **-10: decrease >= 10% -9: decrease 5–<10% -5: decrease 3–<5% -3: decrease 1–<3% -1: decrease <1% 0: no change 1: increase <1% 3: increase 1–<3% 5: increase 3–<5% 9: increase 5–<10% 10: increase >= 10% **Relative magnitude of change 1990–2000 for incomes and median house value: **-10: decrease >= $10,000 -9: decrease $5,000–$10,000 -5: decrease $3,000–<$5,000 -3: decrease $1,000–<$3,000 -1: decrease <$1,000 0: no change 1: increase <$1,000 3: increase $1,000–<$3,000 5: increase $3,000–<$5,000 9: increase $5,000–<$10,000 10: increase >= $10,000.

Housing conditions, except for the median house value, did not change substantially across census tracts and most deviations were within 3% (Figure 4). The median house value decreased by at least $10,000 among 1,771 census tracts.

Several population characteristics remained static among some census tracts primarily because no observation was reported in either census year. For example, 26 census tracts reported no non-Hispanic African-American population in both the 1990 and 2000 censuses, resulting in "No change" in the category (Figure 1).

**Figure 5:**
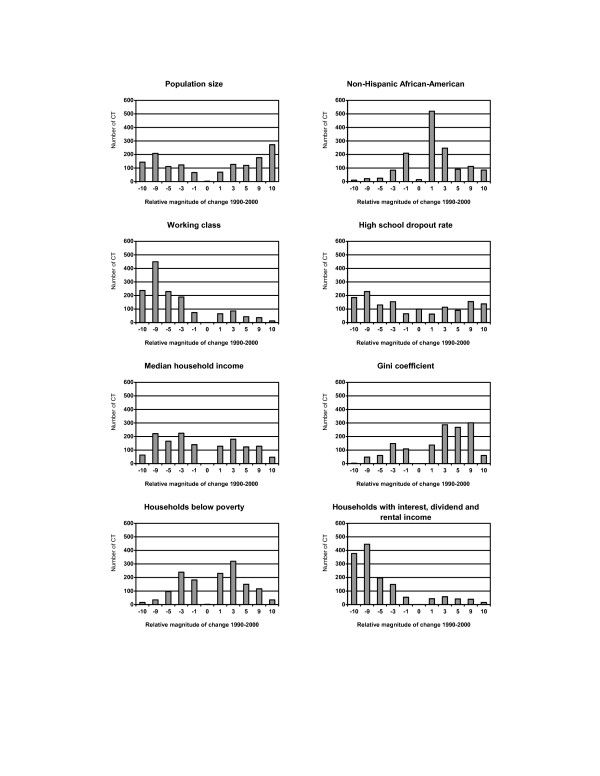
Distribution of changes in selected population characteristics among 1,421 census tracts (CT) without boundary change in NYS exclusive of NYC, 1990–2000. **Relative magnitude of change 1990–2000: **-10: decrease >= 10% -9: decrease 5–<10% -5: decrease 3–<5% -3: decrease 1–<3% -1: decrease <1% 0: no change 1: increase <1% 3: increase 1–<3% 5: increase 3–<5% 9: increase 5–<10% 10: increase >= 10% **Relative magnitude of change 1990–2000 for incomes and median house value: **-10: decrease >= $10,000 -9: decrease $5,000–$10,000 -5: decrease $3,000–<$5,000 -3: decrease $1,000–<$3,000 -1: decrease <$1,000 0: no change 1: increase <$1,000 3: increase $1,000–<$3,000 5: increase $3,000–<$5,000 9: increase $5,000–<$10,000 10: increase >= $10,000.

Subgroup analyses were conducted for 1,421 census tracts without boundary changes between the 1990 and 2000 censuses (Figure 5). Noticeable population shifts were also observed and the patterns were consistent with findings shown in Figures 1, 2 and 3.

### Changes in population characteristics by the rural-urban status

**Figure 6:**
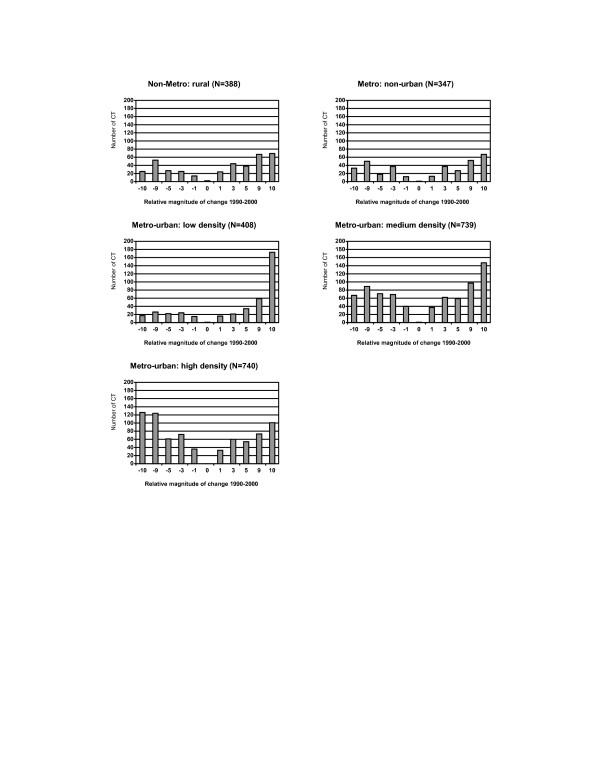
Distribution of changes in population size by the rural-urban status for census tracts (CT) in NYS exclusive of NYC, 1990–2000. **Relative magnitude of change 1990–2000: **-10: decrease >= 10% -9: decrease 5–<10% -5: decrease 3–<5% -3: decrease 1–<3% -1: decrease <1% 0: no change 1: increase <1% 3: increase 1–<3% 5: increase 3–<5% 9: increase 5–<10% 10: increase >= 10%

Population characteristics did not change uniformly across census tracts with respect to their rural-urban status. Urban census tracts with a high population density experienced both sizable losses and gains in population, but other census tracts, especially urban census tracts with low population density, generally increased in size during the decade (Figure 6).

**Figure 7:**
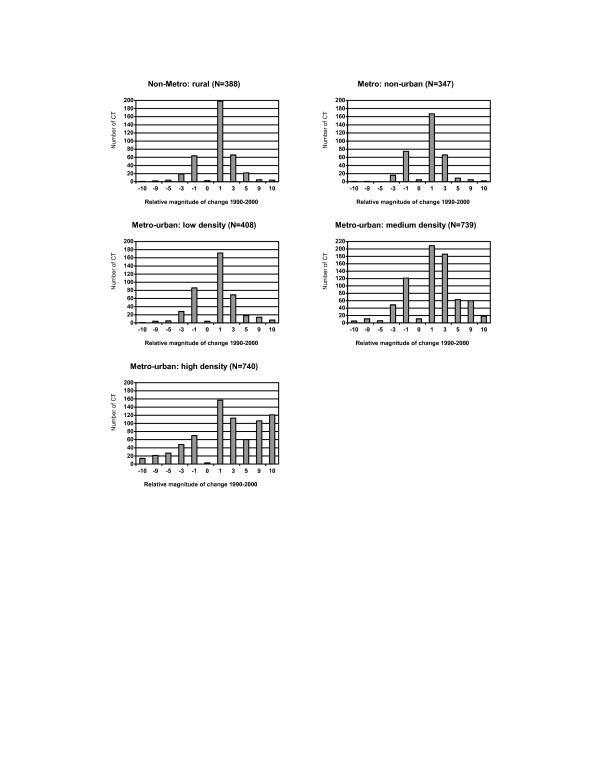
Distribution of changes in non-Hispanic African-American by the rural-urban status for census tracts (CT) in NYS exclusive of NYC, 1990–2000. **Relative magnitude of change 1990–2000: **-10: decrease >= 10% -9: decrease 5–<10% -5: decrease 3–<5% -3: decrease 1–<3% -1: decrease <1% 0: no change 1: increase <1% 3: increase 1–<3% 5: increase 3–<5% 9: increase 5–<10% 10: increase >= 10%

**Figure 8:**
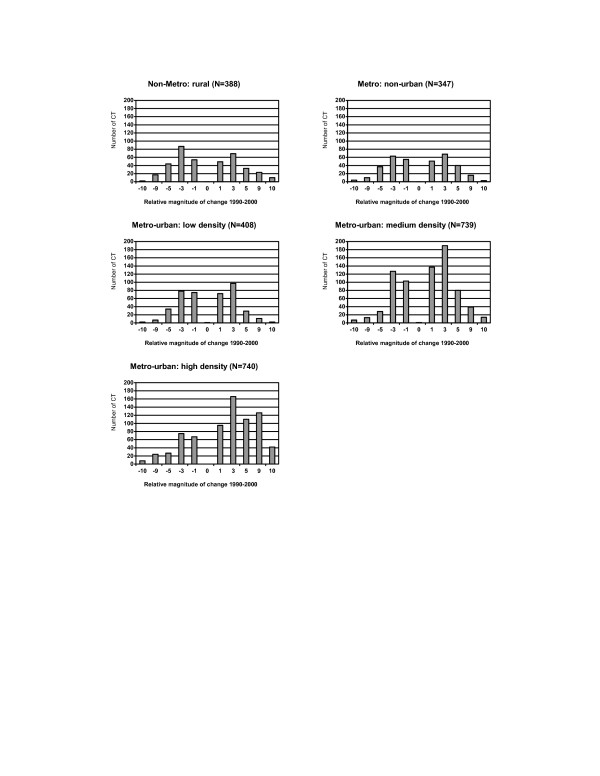
Distribution of changes in household below poverty by the rural-urban status for census tracts (CT) in NYS exclusive of NYC, 1990–2000. **Relative magnitude of change 1990–2000: **-10: decrease >= 10% -9: decrease 5–<10% -5: decrease 3–<5% -3: decrease 1–<3% -1: decrease <1% 0: no change 1: increase <1% 3: increase 1–<3% 5: increase 3–<5% 9: increase 5–<10% 10: increase >= 10%

**Figure 9:**
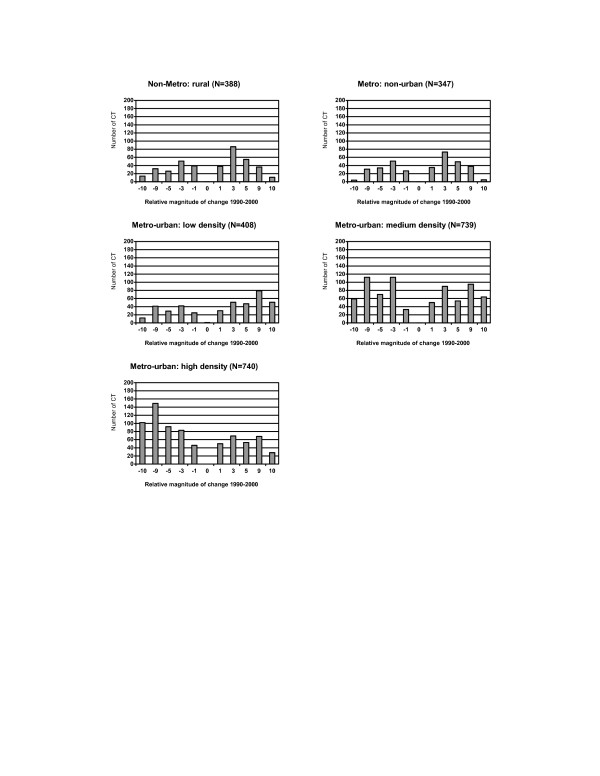
Distribution of changes in median family income by the rural-urban status for census tracts (CT) in NYS exclusive of NYC, 1990–2000. **Relative magnitude of change 1990–2000 for incomes and median house value: **-10: decrease >= $10,000 -9: decrease $5,000–$10,000 -5: decrease $3,000–<$5,000 -3: decrease $1,000–<$3,000 -1: decrease <$1,000 0: no change 1: increase <$1,000 3: increase $1,000–<$3,000 5: increase $3,000–<$5,000 9: increase $5,000–<$10,000 10: increase >= $10,000.

A rural-urban gradient was observed for some characteristics, and differences were more noticeable among urban census tracts. For example, substantial changes in racial distribution and some socioeconomic characteristics, including the high school dropout rate and poverty rates, were evident among urban census tracts with high population densities, while changes were limited to less than 3% among most other census tracts (Figure 7 and Figure 8). In addition, the family income decreased by more than $10,000 for 13.8% of urban tracts compared to 3.5% of rural census tracts (Figure 9).

## Discussion

This study identified substantial shifts in population characteristics that occurred across census tracts in NYS exclusive of NYC between 1990 and 2000. Among the many observed changes, the most important may be those pointing toward greater demographic diversity. Differences in the socioeconomic status over time showed both a growing economy and growing disparities. While there were overall gains in occupational status, education and income, more people live in poverty and fewer households reported having interest, dividend and rental income.

Changes in the population size may be due to aging, which is reflected by the age distribution. The racial-ethnic distributions indicate whites remained the predominant racial group, but African-American accounted for a substantial proportion of the population in some major municipalities and represented a growing demographic statewide. However, the observed increase in the percent of African-Americans was due to both African-American population gains and white population losses, implying that the African-American population became more concentrated in some census tracts, especially those located within densely populated urban areas.

Many findings in this study were consistent with previous research: more female-headed families [27,28], less working class population [27], improved educational status [27,29], higher incomes [23,25-27,29], increased income inequality [22,23,25,27] and higher poverty rates [26,27]. However, the decreased median house value was not in agreement with previous findings [27]. In addition, this study elucidates the pattern of dramatic bi-directional census tract changes. Over the ten-year period, almost all census tracts experienced population shifts, and some census tracts had a greater than 10% change in demographic and socioeconomic characteristics. Various patterns of change occurred across population characteristics as well as along the rural-urban gradient.

Because the magnitude of change in one decade may not be ignorable, this study raises concerns about using population estimates, particularly for subgroup estimates (e.g., age, race-ethnicity), measured at one point in time as baseline when assessing disease trends over time. Numerous ecologic studies have shown that baseline population socioeconomic characteristics, the main exposure of interest, were inversely associated with population mortality or disease incidence rates [2,4,15-19]. Multilevel studies, usually modeling baseline population characteristics at the second level to assess if they were directly associated with individual-level risk of disease, also indicated that people living in deprived communities were more likely to develop adverse health outcomes in a defined time period [3,5,10]. However, the possible causal inference between the social environment and individual's risk of disease remained unclear. Including the effect of population shift measured at multiple time points in longitudinal studies may help to more fully characterize the social context and understand its role on disease development.

Identifying population changes is not only of value for public health research, but also of importance for public health planning. In order to efficiently allocate and administer federal and state funding to local jurisdictions, Community Health Assessments are conducted periodically in NYS to describe changes over time of both population characteristics and disease trends [38]. Results from community assessments help decision-makers redirect program efforts to communities as needed to facilitate development of disease intervention strategies.

It should be noted that coping with the redefinition of census tract boundaries is an important challenge in longitudinal research, and complicated the comparisons in this study. In previous studies, variable census boundaries were not a major issue for one of several reasons: large census units with stable boundaries were used [22,26,27], units with boundary changes were excluded from the analysis [12,23], or the boundary changes were mainly due to straightforward splitting of existing tracts [29]. In contrast, most census tracts in NYS with boundary changes were redrawn into new tracts instead of being simply merged or split. In the NCDB, an advanced geographic information system was used to overlay the 2000 boundaries with the 1990 boundaries to determine the population weight for data conversion [31]. However, using different methods to manage changes in census tract boundaries [39] might lead to alternate conclusions regarding population trends. To test this possibility, we conducted additional analyses for 1,421 census tracts without boundary changes between the 1990 and 2000 censuses and found that changes in population characteristics persisted even among these census tracts.

Because this study was conducted at the census tract level, population patterns may not be generalized to other census units, such as county or the census block group. As expected, less variation in population characteristics was seen at the county level (data not shown); data were not examined at the census block group level due to the lack of detailed boundary change information for block groups. All census tracts were given equal weight in the analyses because we were interested in assessing changes in "neighborhoods" and did not want to hide potentially interesting changes in small neighborhoods by weighting. Stratified analysis by the rural-urban status was conducted but may not be able to fully adjust the difference in population sizes across census tracts. Undercounting populations in the census is another possible limitation for studying population changes. In the 1990 census, minority populations, especially African-American, were more likely to be under-sampled than whites [40]. In 2000, there was a substantial reduction in the undercounting rates nationwide and the Census 2000 response rate for NYS (63%) was slightly higher than that in the Census 1990 (62%) [41-43], so the increases in the minority populations observed in this study may be partially due to overall improvements in population sampling. Missing or inconsistent information on population characteristics may also affect the comparison across censuses. The item nonresponse rates in Census 2000 were higher than in Census 1990 because, unlike Census 1990, no extensive follow-up was conducted in Census 2000 for people with substantial missing data [44]. In Census 2000, the nonresponse rates were less than 5% for population demographics, but greater than 10% for some items related to socioeconomic status and housing conditions (e.g., income, house value). The imputation process developed by the Census Bureau to assign values for missing or invalid items for the 2000 Census shows a "reasonably high quality" [44].

In our study, only single measures of population characteristics were examined, and indices, which are commonly used in studying social determinants of health, were not included primarily because they are subject to variation across studies [5,9-11,17,18,20,33]. In addition, an index derived from several variables may exaggerate or mask the changes for those variables. However, the authors still recommend examining the change in an index for longitudinal research based on indices.

Because census data are released every decade, it is possible that substantial population changes have already occurred during the current decade. The American Community Survey (ACS), the largest random sample household survey developed by the Census Bureau since 1996 to collect population and housing data on an annual basis, produces estimates of population characteristics for geographic areas with populations of 65,000 or more, and will be able to provide three-year or five-year average population estimates for smaller areas including the census tract and census block group after 2007 [45]. Some commercial companies can also provide single-year population estimates at various geographic levels using data released by the Census Bureau for public health researchers to evaluate population shifts.

Dynamic effects of population characteristics on health outcomes have not been commonly studied, and the statistical models used for analyzing effects of population change varied across studies [11,12,22,23]. Further research is needed to assess if the statistical methods developed for individual-level longitudinal studies can be applied to ecologic studies or multilevel analysis involving population shifts [46].

## Conclusion

In summary, our findings illustrate that regarding most population characteristics analyzed in this study, few remained static, and some population characteristics changed considerably. In order to correctly interpret secular trends of disease at the ecologic level, researchers should be cognizant of and account for dynamic changes in population characteristics that influence the health outcome of interest and multiple factors should be included in the study to capture such population patterns.

## Abbreviations

NYS: New York State

NYC: New York City

NCDB: Neighborhood change database

CPI: Consumer price index

SD: Standard deviation

PPSM: People per square mile

CT: Census tracts

## Competing interests

The author(s) declare that they have no competing interests.

## Authors' contributions

PD designed and performed the statistical analysis; FBC and PO participated in the study design; LAM participated in the study design and supervised the conduct of the study; and all authors contributed to the write up of the manuscript. All authors read and approved the final manuscript.
